# Intracellular Delivery of DNA and Protein by a Novel Cell-Permeable Peptide Derived from DOT1L

**DOI:** 10.3390/biom10020217

**Published:** 2020-02-02

**Authors:** Jingping Geng, Xiangli Guo, Lidan Wang, Richard Q. Nguyen, Fengqin Wang, Changbai Liu, Hu Wang

**Affiliations:** 1 Department of Pathology and Immunology, Medical School, China Three Gorges University, Yichang 443002, China; gjp081188@outlook.com (J.G.); biomed-xiangli-guo@outlook.com (X.G.); wang473119037@outlook.com (L.W.); 2 Hubei Key Laboratory of Tumor Microenvironment and Immunotherapy, China Three Gorges University, Yichang 443002, China; wfq1248869512@outlook.com; 3 Institute for Cell Engineering, Johns Hopkins University School of Medicine, Baltimore, MD 21205, USA; nguyen.richard15@gmail.com

**Keywords:** cell-permeable peptides (CPPs), plasmid, transfection, protein delivery

## Abstract

Cellular uptake and intracellular release efficiency of biomacromolecules is low because of hurdles in the cell membrane that result in limited access to intra-cellular targets with few functional effects. Cell-penetrating peptides (CPPs) act as cargo delivery vehicles to promote therapeutic molecule translocation. Here, we describe the novel CPP-Dot1l that not only penetrates by itself, but also mediates cargo translocation in cultured cells, as confirmed by fluorescence microscopy and fluorescence spectrophotometry. We conducted cytotoxicity assays and safety evaluations, and determined peptide-membrane interactions to understand the possible pathway for cargo translocation. Additional nucleic acid and covalently conjugated green fluorescence protein (GFP) studies mediated by CPP-Dot1l were conducted to show functional delivery potential. Results indicate that CPP-Dot1l is a novel and effective CPP due to its good penetrating properties in different cell lines and its ability to enter cells in a concentration-dependent manner. Its penetration efficiency can be prompted by DMSO pretreatment. In addition, not only can it mediate plasmid delivery, but CPP-Dot1l can also deliver GFP protein into cytosol. In conclusion, the findings of this study showed CPP-Dot1l is an attractive pharmaceutical and biochemical tool for future drug, regenerative medicine, cell therapy, gene therapy, and gene editing-based therapy development.

## 1. Introduction

Promising therapeutic bio-macromolecules such as nucleic acids (DNA and/or RNA) have become widely recognized in the field of reprogramming-based regenerative medicine [1,2,3] and gene editing [4] for drug development research. However, semi-permeable cellular membranes serve as a barrier, which makes it challenging for nucleic acids to enter the cytoplasm because of their size and overall hydrophilic nature [4].

Drug delivery vehicles ranging from polymers (including stimuli responsive polymers) and non-organic nanoparticles to liposomes are commonly used. Some of them are under preclinical and clinical studies to evaluate their suitability for disease treatment. To date, three iron oxide nanoparticles (Feridex, GastroMARK, and Feraheme) have been approved by the Food and Drug Administration (FDA) [5]. However, it is worth noting that two of them have been withdrawn from the market. Although approaches such as microinjections and electroporation have also been developed for drug delivery, these approaches have limitations, including poor efficiency in vivo, poor tissue or cell specificity, and low bioavailability [4]. One of the main advantages of viral vectors is that genetic material delivery approaches 95–100% efficiency. Disadvantages include insertional mutagenesis and the potential for the activation of latent diseases, which impacts immunogenicity and inflammation, becoming an obstacle limiting their success in clinical trials and further applications [4,6]. Considering these limitations, investigators worldwide must continue to build better delivery systems. This prompted us to explore the potential peptide-based delivery systems.

Cell-penetrating peptides (CPPs), also known as protein transduction domains (PTDs), can pass through tissue or cell membrane with low cytotoxicity and have been found to be very effective in transporting non-covalently and/or covalently conjugated bioactive molecules [7,8]. The existing CPPs such as Transactivator of transcription (TAT) and fusogenic hemagglutinin peptide HA2 have the capacity to overcome many intra- or extracellular barriers. While delivery efficiency limitation is still the main obstacle to the development of drug delivery [9,10], the immunogenicity from an additional HA2 fusion peptide may lead to the activation of immune response in vivo [11].

Although numerous research efforts show CPPs as potential delivery tools, CPP-based nucleic acid delivery applications are limited and their efficiency is often insufficient [12,13]. To improve CPP-based DNA delivery ability, various approaches have been developed, such as optimization of delivery conditions and peptide modification [14,15]. While poor efficiency is still the major disadvantage of their application, some penetration enhancers have been identified [9,16,17], creating interest in identifying CPPs and attracting the attention of researchers in the field of drug delivery [6,18,19,20].

We report the identification of a 24-residue sequence from the Dot1l protein, an arginine-rich primary structure that serves as a novel human origin protein. Not only does it have penetration properties, but Dot1l also acts as a carrier for GFP proteins, resulting in efficient translocation. In addition, it is worth mentioning that CPP-Dot1l does not need to be covalently linked to the attached nucleotides to get them into the cell. This attribute becomes an asset in a more convenient vehicle for nucleic acid delivery. Our results demonstrate that the human gene DOT1L origins of CPP-Dot1l provide an option for non-viral-based nucleic acid delivery in next-generation gene or cell therapy.

## 2. Material and Methods

### 2.1. Peptide Synthesis, Plasmid DNA and Protein Purification, Cells, and Cell Culture

Dot1l peptides (FITC-(Acp)-KARKKKLNKKGRKMAGRKRGRPKK) were synthesized by ChinaPeptides (Shanghai, China) with N-terminal fluorescein isothiocyanate (FITC) labeling at the reversed phase analytical high-performance liquid chromatography level (>99% purity). A labeled nonsense NCO (FITC-(Acp)-KALGISYGRKK) peptide sequence was used as negative control: Synthesized peptides were dissolved with phosphate-buffered saline (PBS), diluted to 0.5 mM in solution and stored at −20 °C until use.

Well-constructed plasmid DNA (pDNA) was amplified using DH5α strain of *Escherichia coli* and the pDNA was extracted using TIANperp Rapid Mini Plasmid Kit (Tiangen Biotech, Beijing, China) based on the manufacturer’s recommendations. The quality of plasmid DNA was examined and then stored at −20 °C until use.

pET15b-GFP-Dot1l plasmid DNA was also well-constructed and recombinant fusion protein was produced in the BL21 (DE3) strain of *E. coli*. Induction of GFP-CPP-Dot1l fusion protein expression was conducted by 100 µM isopropyl-β-D-thiogalactopyranoside (IPTG) at 20 °C for 16 h. Cells were harvested by centrifugation and resuspended in lysis buffer containing 20 mM Tris pH 8.0 and then lysed by sonication. Fusion protein was purified using Ni-NTA affinity chromatography (Qiagen) and dialyzed into PBS and concentrated by ultrafiltration (Millipore).

Human breast cancer cell lines MCF7, human hepatocellular carcinoma-derived HepG2, human cervical carcinoma-derived Caski, mouse melanoma cell B16, and rat hepatic stellate cell line HSC-T6 cells were routinely maintained in our lab. All cell lines were grown in Dulbecco’s modified Eagle’s medium (DMEM) Plus with 10% heated-inactivated fetal bovine serum (FBS) and 1% penicillin–streptomycin at 37 °C and 5% CO_2_.

### 2.2. Bioinformatic Assay

I-TASSER server (http://zhanglab.ccmb.med.umich.edu/I-TASSER) and Pepstr (peptide tertiary structure) were used to generate three-dimensional (3D) structures of Dot1l peptide [21]. To validate the above prediction, molecular dynamic, energy minimization, and modeled structures were further confirmed by the Volume Area Dihedral Angle Reporter (VADAR) web server [22]. The Molegro molecular viewer was used to present the 3D structure, energy map, surface electrostatics, and surface hydrophobicity of the CPP-Dot1l peptide. To further identify possible errors in the 3D structures predicted, validation was conducted by ProSA-web server (https://prosa.services.came.sbg.ac.at/prosa.php) [23] and included overall model quality (Z score) checking and local model quality (residue-wise energy plot) checking. The PPM web server (http://opm.phar.umich.edu/) [24] was used to predict the orientation of membrane permeation and depths of the CPP-Dot1l peptide relative to the membrane binding affinities. The MCPep server (http://bental.tau.ac.il/MCPep/) [25] was also used to conduct Monte Carlo (MC) simulations of Dot1l, hPP3, and hPP10 peptides relative to the lipid membrane bilayer, which provided discrimination between the surface mechanism of action and the transmembrane (TM).

### 2.3. Cellular Uptake and Fluorescent Microscopy

All types of cells were suspended in 0.5 mL media and seeded at a density of 1.5 × 10^5^ cells/well on a 24-well plate for 24 h. After washing with PBS twice, the indicated cells were incubated with indicated concentrations of FITC-CPP-Dot1l, FITC-NCO peptides, or purified protein in 0.5 mL serum-free media/well for 1 h. After incubation, cells were rinsed three times in PBS and then imaged by fluorescence microscopy (Nikon, Tokyo, Japan). To evaluate the cellular uptake efficiency of indicated treatment, the cellular uptake of indicated concentration of CPP was quantified with multimode spectrophotometry (Tecan, Mannedorf, Switzerland). The incubation and wash procedure were the same as above. Cells were then lysed with 0.3 mL/well lysing buffer (0.1 M NaOH) for 10 min and centrifuged at 14,000 rpm for 5 min. The fluorescence intensity in the supernatant was read using Multimode spectrophotometry (Tecan, Mannedorf, Switzerland) at 485 nm excitation and emission at 535 nm. Protein concentration of supernatant was examined by the Bradford protein assay kit following the manufacturer’s recommendation. The fluorescence of cellular uptake is expressed as fluorescence intensity per mg of total cellular protein. Experiments indicated in the text were repeated at least three times.

The uptake mechanism of Dot1l in MCF7 cells was examined through endocytosis inhibitors. Peptide incubation at indicated different temperatures was measured to investigate the influence of temperature on peptide internalization (physical means of inhibition of endocytosis). Peptide incubation with different kinds of inhibitors, such as serum (10% FBS), NH_4_Cl (50 μM), NaN_3_ (40 μM), heparin (50 μg/mL), Methyl-β-cyclodextrins (MβCDs) (5 mM), wartmannin (5 μM), and hyperosmotic sucrose (0.45 M) was also examined. The uptake efficiency was detected using the same protocol as the one shown above.

### 2.4. Hemolysis Assay

To examine the membrane integrity, red blood cells (RBC) were used to conduct hemolytic analysis. After separation of RBCs by centrifugation for 10 min at 500 rpm, RBCs were collected and carefully washed with PBS. A 20% *v/v* RBC suspension was used for further experiments. In a typical experiment, 25 μL of RBC suspension were added to 225 μL peptide dilutions at different concentrations. Following 2 h of incubation, samples were centrifuged (500 rpm, 5 min) to discard cells and the membrane fragment. Supernatant samples (50-μL aliquots) were transferred to a clear 96-well plate and hemoglobin absorbance was read at 450 nm. Experimental design contains negative controls and positive controls (RBCs treated with 0.1% Triton X-100).

### 2.5. Cytotoxicity Assay

HSC-T6 and MCF7 cells were seeded at a density of 8000 cells/well in 96-well culture plates overnight before incubation. The cells were washed with PBS and were treated with Dot1l or Dot1l/pDNA complexes of different concentrations at the indicated times. After rinsing with PBS, 20 μL of 5 mg/mL MTT in PBS solution were added to 80 μL of serum-free media and incubated for 4 h. After that, the culture medium was discarded and 150 μL of dimethyl sulfoxide (DMSO) were added into each well to dissolve the formazan crystals. The absorbance of DMSO-dissolved solution was read in a Multiskan Spectrum (Thermo Fisher Scientific, Waltham, MA, USA) reader at 490 nm.

### 2.6. Lactate Dehydrogenase Leakage Assay

Lactate dehydrogenase (LDH) assay was conducted to measure the release of lactate dehydrogenase from damaged cells. Cells were seeded at a density of 1.5 × 10^5^ cells/well to 24-well plates for overnight culture and peptides at indicated concentrations were added as described above. After 1 h incubation, 50 μL of cell-free supernatant were collected and added to each well, including controls and cell-free wells filled with 50 μL of LDH assay buffer. Reaction was conducted at room temperature (RT) for 10 min according to the manufacturer’s recommendations and the Optical Density (OD) was read in a Multiskan Spectrum (Thermo Fisher Scientific) plate reader at 570 nm.

### 2.7. Gel Retardation Assay

The plasmid DNA condensation capability of CPP-Dot1l was examined by agarose gel retardation assay. Agarose gel separation was performed in 1× Tris-acetate-EDTA (TAE) buffer. Dot1l peptide was gently mixed with pcDNA3.1-GFP (1 μg) at indicated nitrogen to phosphate ratios (N/P) ratios in Milli-Q water or 50% serum at RT for 30 min. Afterwards, the peptide/pDNA mixture was separated by 1% agarose gel. Images were captured using the Kodak Gel Logic 2200 Imaging System.

### 2.8. Zeta-Potential and Particle Size Measurement

The Dot1l/pDNA complexes with the indicated N/P ratio were mixed in accordance to the protocol established [26,27]. The mean zeta potential and average diameter of the peptide/pDNA complexes were examined by Zetasizer (Zetasize-Nano ZS90; Malvern Instruments, Worcestershire, UK) and data analysis was performed with Zetasizer software 6.30.

### 2.9. Peptide-Mediated Transfection

HSC-T6 and MCF7 cells (4 × 10^4^ cells/well) were seeded onto 24-well plates 24 h before transfection; then, they were pretreated with 5% dimethyl sulfoxide (DMSO) for 30 min. CPP-Dot1l/pDNA complexes at indicated the N/P ratio were gently added to the cells with 300 μL serum-free media. After 4 h incubation, 300 μL of full growth media were added into the well and afterwards were cultured for 24 or 48 h. The peptide-based transfection efficiency was examined under fluorescence microscope (Nikon) after PBS washing. TurboFectin (OriGene, Beijing, China) was used as a positive transfection reagent.

### 2.10. Western Blotting

After fusion GFP or GFP-Dot1l protein treatment and three-time wash step in cold PBS, cells were lysed by cold 0.1% Triton X-100 lysis buffer with the supplemented protease inhibitor phenylmethylsulfonyl fluoride (PMSF). Cell lysates were incubated 30 min on ice. Cell lysates were centrifuged at 12,000 rpm for 20 min, supernatant was collected, and its concentrations were quantified using the BCA Protein Assay Kit following the manufacturer’s recommendations. Protein samples were separated by 10% sodium dodecyl sulfate polyacrylamide gel (SDS-PAGE), followed by transfer onto a polyvinylidene difluoride (PVDF) membrane. After PVDF membrane blocking in blocking buffer (5% non-fat dry milk in TBST (0.1% Tween-20 in Tris-buffered saline (TBS))) for 1 h at RT, primary antibody anti-GFP (Rabbit polyclonal, Cell Signaling Technology; 1:1000) was used for immunoreactions overnight at 4 °C. After washing the PVDF membrane with TBST three times, secondary antibody labeled with horseradish peroxidase conjugate (Solarbio; 1:3000) was added and incubated for 1 h at RT. Anti-β-actin-horseradish peroxidase (HRP) (Santa Cruz Biotechnology; 1:1000) was used as loading controls of immunoreactions. Chemical reaction light signals were detected by Clinx ChemiScope 3000 mini using enhanced chemiluminescence (ECL)-detecting reagent.

### 2.11. Flow Cytometry Analysis

Cells and Dot1l peptide (10 μM), following the same method as described before, were plated with treatment in a six-well plate format. Cells were then harvested and pelleted in washing steps with PBS three times by centrifuging for 5 min at 1023 rpm. The supernatant was removed, and cells were resuspended in PBS. Cells were then fixed in 70% cold ethanol in PBS for 30 min. After washing three times with PBS, the fixed cells were stained with 0.5 mL PI/RNase A solution or with the Annexin-V/PI Apoptosis Detection Kit (Elabscience, Wuhan, China), following the recommended methods. Quantification of cell cycle states or cell apoptosis was acquired by fluorescence of each sample in BD FACSVerse™ flow cytometer.

### 2.12. Statistical Analysis

All present control and experimental values are expressed as means ± standard deviation (SD). Significance analysis was conducted using GraphPad software Prism 7.0 (GraphPad Software, San Diego, CA, USA). Differences of *p* < 0.05 were considered significant.

## 3. Results

### 3.1. The Modeled Structure of Dot1l Peptide

**Figure 1:**
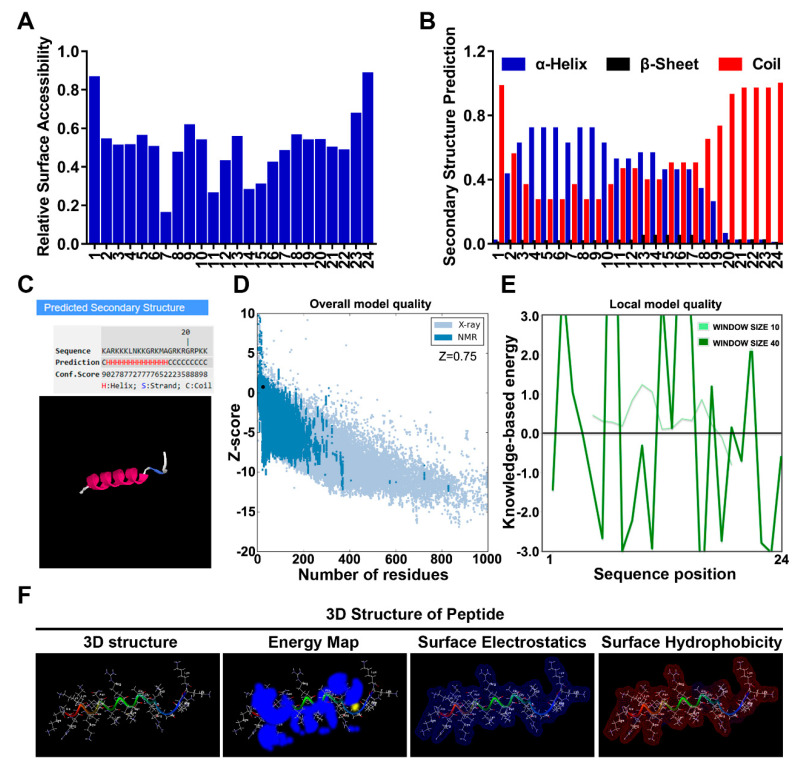
Dot1l peptide structure prediction. (**A**) Relative surface accessibility prediction of Dot1l peptide by the NetSurfP web server. (**B**) Dot1l peptide secondary structure prediction on the NetSurfP web server. (**C**) Structural model of Dot1l peptide predicted on the I-TASSER server. (**D**) ProSA-web z score plot for the Dot1l peptide 3D structure generated from the I-TASSER server. (**E**) ProSA local model quality/residue-wise energy plot of the Dot1l peptide 3D structure generated from the I-TASSER server. (**F**) Representation tridimensional structure, energy map, surface electrostatics, and surface hydrophobicity of Dot1l peptide.

The fundamental feature determining the penetrating of a CPP is its 3D structure; therefore, peptide structure prediction by multistep algorithm was used to model the structure of Dot1l peptide. Relative surface accessibility (Figure 1A) and secondary structure prediction (Figure 1B) of the residue in Dot1l peptide were analyzed using NetSurfP web server. The alpha helix-shaped structure predicted by the I-TASSER server (Figure 1C) was consistent with the helix prediction by the NetSurfP web server. The prediction quality and potential errors of the Dot1l tridimensional structure modeled by the I-TASSER server were examined using ProSA-web. The z-score from the prediction initial entrance model reached 0.75 (Figure 1D). The ProSA local model quality/residue-wise energy were also evaluated (Figure 1E) and indicated good quality of the initial model. Moreover, the 3D structure, energy map, surface electrostatics, and surface hydrophobicity of Dot1l are presented (Figure 1F).

### 3.2. Penetrating Property of Dot1l Peptide

**Figure 2:**
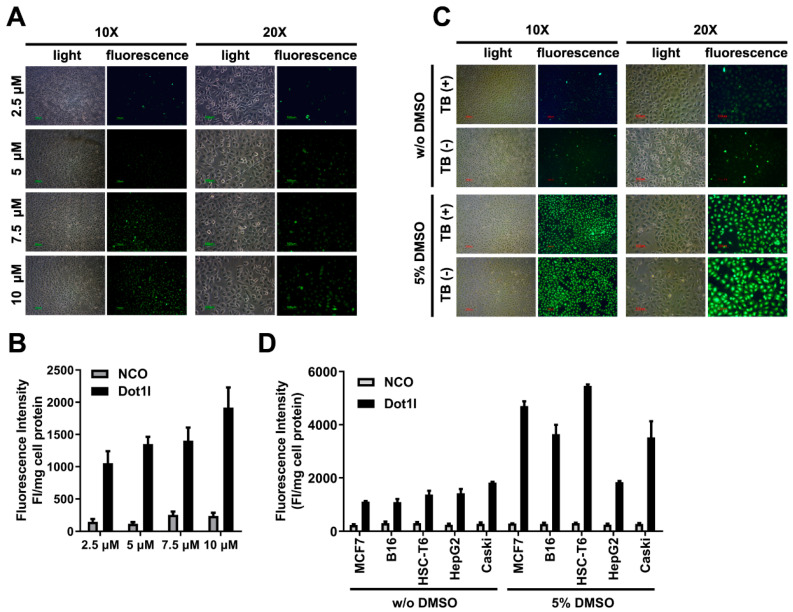
Fluorescence labelled Dot1l peptide penetration in cultured cells. (**A**) Fluorescence microscopy of fluorescein isothiocyanate (FITC)-labeled Dot1l peptide with different concentrations. (**B**) Quantization analysis of FITC-labeled Dot1l peptide corresponding to fluorescence microscopy with different concentration measurements. The statistical analysis is shown in Supplementary Figure S1A. (**C**) Fluorescence microscopy of FITC-labeled Dot1l peptide with or without trypan blue incubation in the DMSO-pretreated or control group. (**D**) FITC-labeled Dot1l peptide penetration in different cell lines (MCF7, B16, HSC-T6, Caski, and HepG2) with or without DMSO pretreatment. The statistical analysis is shown in Supplementary Figure S1B,C. Cell lysate fluorescence intensity was adjusted by protein concentration examined by Bradford assay.

Following the documented procedure and protocol for the penetration property of CPP using in vitro assay [9,26,27], MCF7 cells incubated with FITC-labeled Dot1l peptide (5 µM) for 1 h, without DMSO pretreatment, were examined by fluorescence microscopy. Fluorescence imaging was conducted to examine Dot1l peptide penetrating ability at indicated concentrations (from 2.5 to 10 µM). The fluorescence was apparently increased as its concentration increased (Figure 2A) and a quantitative evaluation (Figure 2B and Supplementary Figure S1A) of Dot1l penetration was also performed. Arginine played a vital role in CPP penetration, and while Dot1l is an arginine-rich peptide, the major pathway of Dot1l is through direct translocation. Even if we scrambled peptides, they would still have enough arginine to penetrate into the cells; therefore, we chose nonsense NCO peptides as our control and set the baseline of non-translocation to depend on NCO treatment. The penetrating efficiency of Dot1l peptide was increased significantly compared with non-sense peptide NCO group. Trypan blue was used to eliminate the signal at the cell periphery [28]; however, the fluorescence signal persisted, which indicated intracellular penetration of the Dot1l peptide. As in the 5% DMSO pretreatment group, the intracellular fluorescence signal was highly increased compared with the non-DMSO control group (Figure 2C and Supplementary Figure S2A). In addition, the penetration efficiency of longer incubation time of Dot1l was not promoted (Supplementary Figure S2B). To evaluate cell-type specificity of Dot1l penetration, different cell lines’ (MCF7, B16, HSC-T6, Caski, and HepG2) uptakes of Dot1l peptide were evaluated (Figure 2D, Supplementary Figures S2C and S1B), suggesting that Dot1l peptide in the non-DMSO pretreatment condition could penetrate into all different cell lines efficiently without apparent cell line specificity. After 5% DMSO pretreatment, Dot1l peptide penetration significantly increased (Supplementary Figure S1C); these data are consistent with our previously published findings [9]. Moreover, the penetration efficiency of different CPPs was compared (Supplementary Figure S2D), and the penetration efficiency of Dot1l was weaker than hPP10, but significantly higher than that of TAT and MT23, a new B16 targeting peptide characterized in a previous study [6].

### 3.3. Different Conditions on the Penetrating Property of Dot1l Peptide

**Figure 3:**
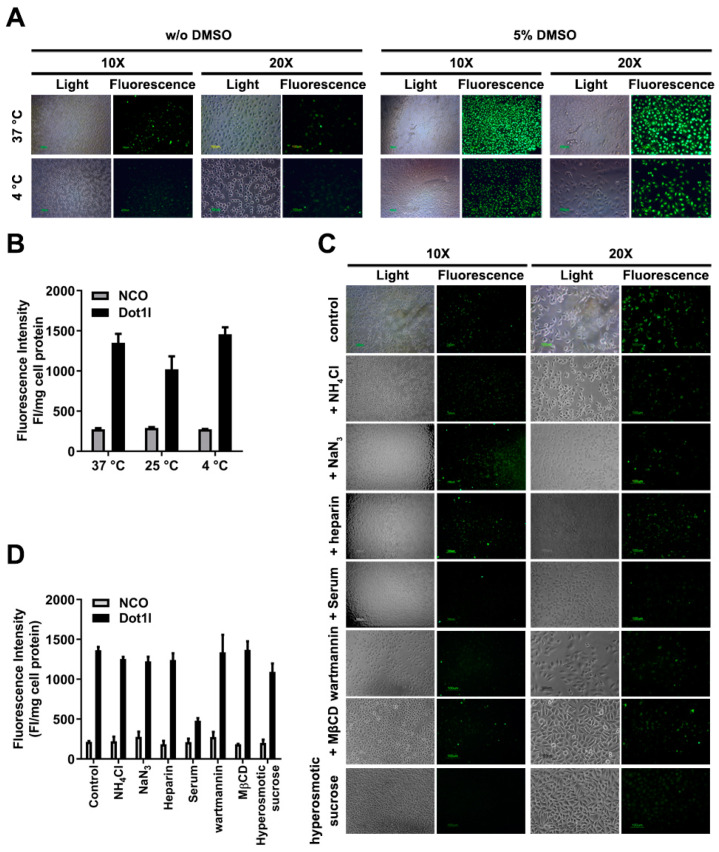
Effects of Dot1l peptide penetration under different conditions. (**A**) Fluorescence microscopy of FITC-labeled Dot1l peptide at 37 °C and 4 °C. (**B**) Quantization of Dot1l peptide penetration in MCF7 cells at different temperatures. Data are presented as means ± SEM (*n* = 3). The statistical analysis is shown in Supplementary Figure S1D. (**C**) Fluorescence microscopy of different inhibitors’ exposure on Dot1l peptide penetration. (**D**) Suppression of different inhibitors’ exposure on Dot1l peptide penetration. Data are presented as means ± SEM (*n* = 3). The statistical analysis is shown in Supplementary Figure S1E. Cell lysate fluorescence intensity was adjusted by protein concentration examined by Bradford assay.

Besides experimental factors (cell type and incubation time) that affect the uptake mechanism, we also examined whether temperature directly influences the penetrating property of Dot1l. As shown in Figure 3A, our results revealed that fluorescence intensity in the 37 °C group was significantly higher than that of the 25 °C group; however, there were no apparent differences when compared with the 4 °C group (Figure 3B and Supplementary Figure S1D). These data suggest that Dot1l penetration may partially involve temperature-dependent cellular entry pathways, although some references in the field highlighted an energy-independent contribution to CPP uptake [29,30,31]. To further understand the precise mechanism of Dotl1 peptide uptake, selective and specific interventions in endocytosis studies were conducted. Cellular uptake efficiency of Dot1l peptide was examined with or without internalization inhibitors, as shown in Figure 3C. Dot1l internalization in the presence of serum containing incubation medium was blocked. Moreover, Dot1l internalization was not blocked by 40 µM of oxidative phosphorylation inhibitor sodium azide (Figure 3C,D, Supplementary Figure S1B) and 50 mM of ammonium chloride (anti-acidification agent of endocytic vesicles). As a cell-surface endocytosis receptor, heparan sulfate proteoglycans (HSPGs) serve as the initial anchoring site for many known CPPs via electrostatic interactions between negatively charged sulfates/carboxylate groups and positive charged residues (Arg, Lys, and to some extent His). We found that 50 μg/mL of heparin (HSPG competitor) could not block Dot1l uptake efficiently (Figure 3C,D, Supplementary Figure S1B), which suggested that Dot1l penetration may be endocytosis-independent. Additionally, more specific inhibitors were further used to examine the mechanism of Dot1l uptake. When compared with the control group, there were no significant differences in Dot1l internalization in the groups treated with 5 mM MβCD (an inhibitor of lipid raft-mediated endocytosis), 5 μM wartmannin (a PI-3 kinase blocking agent that indirectly affects receptor mediated endocytosis), and 450 mM sucrose (a hyperosmotic sucrose that inhibits clathrin-dependent endocytosis by preventing clathrin and adaptors from interacting [32]). This suggests that lipid raft-mediated endocytosis may not mediate intracellular delivery of Dot1l, although receptor-mediated endocytosis may be slightly involved. Therefore, we found that endocytosis may not be the major pathway involving the translocation of Dot1l peptide, even though the endocytosis pathway can mediate CPP uptake [30].

### 3.4. Dot1l Peptide-Membrane Interaction Prediction

**Figure 4:**
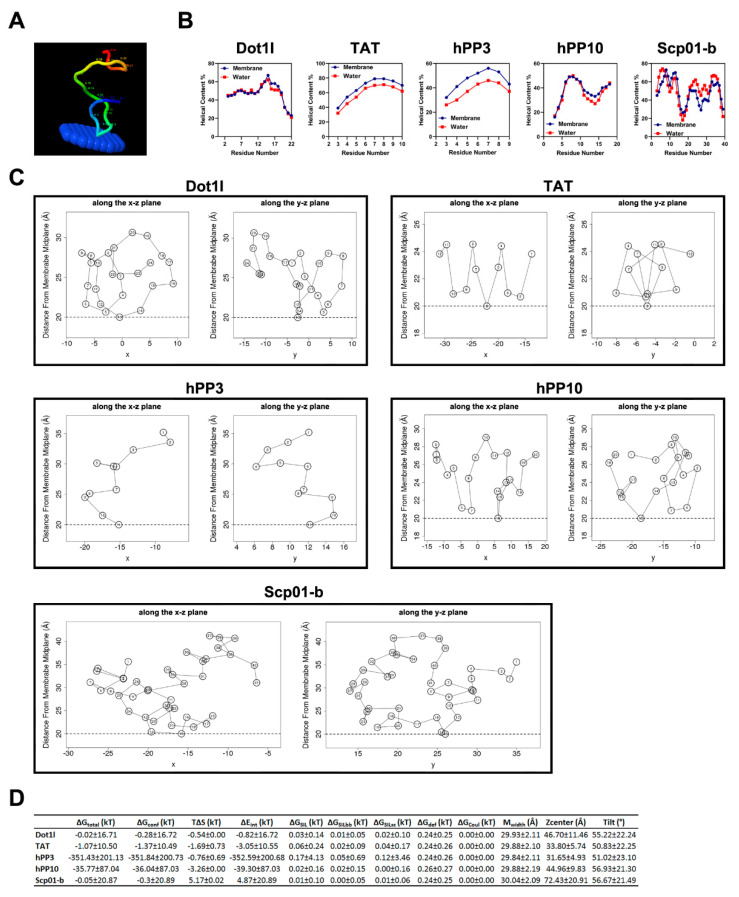
Dot1l peptide–membrane interaction prediction by the PPM and MCPep web server. (**A**) Dot1l peptide–membrane interaction prediction by PPM web server. (**B**) Different CPP/secondary structure propensity prediction in water and lipid bilayers (membrane) environments using the MCPep web server. (**C**) The average location of the amino acids of different CPPs (Dot1l, hPP3, hPP10, and Scp01-b) in the membrane on the surface. The horizontal dashed lines indicate the location of the phosphate groups of the lipid polar heads (membrane includes 30% charged lipids). (**D**) Thermodynamic characteristics for Dot1l peptide in surface configurations (all values presented as means ± SE).

To further investigate the penetration of Dot1l peptide, the interaction between Dot1l peptide and membrane was predicted using PPM web server and MCPep server. As our previous paper has shown, well-known CPP-TAT [9], hPP3 [19], and hPP10 [20,26] can partially be inserted into the membrane predicted by the PPM web server [27], similar to Dot1l peptide’s weak property of membrane insertion (Figure 4A). The MCPep server, a computational tool for the prediction of peptide (secondary structure) occurrence in lipid bilayers and aqueous environments, was used for further prediction of interactions between Dot1l peptide and membrane. Dot1l, hPP10, and Scp01-b presented α-helical conformations in both lipid bilayers and aqueous environments (Figure 4B) while TAT and hPP3 tended to acquire a random coil conformation. However, in membranes, unlike Dot1l, hPP10, and Scp01-b, TAT and hPP3 tend to form α-helical conformations. The simulation predicts weak membrane association without any insertion for Dot1l peptide (Figure 4C). Additionally, thermodynamic characteristics for Dot1l, hPP10, Scp01-b, TAT, and hPP3 in surface configurations (Figure 4D and Supplementary Figure S3) suggested that Dot1l peptide interacted with membranes but could not enter into the membrane stably.

### 3.5. Cytotoxicity Evaluation of Dot1l Peptide

**Figure 5:**
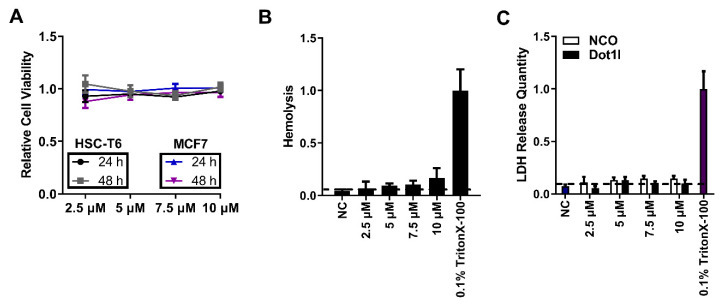
Cytotoxicity evaluation on Dot1l peptide. (**A**) Cell viability of HSC-T6 and MCF7 cells treated with Dot1l peptide at indicated concentrations examined after 24 and 48 h by MTT assay. Data are presented as means ± SEM (*n* = 3), *p* > 0.05, two-way analysis of variance (ANOVA) with Bonferroni’s multiple comparison test. (**B**) Cell membrane integrity examined by hemolysis assay after different concentrations of Dot1l peptide treatment; 0.1% Triton X-100 used as positive control. Data are presented as means ± SEM (*n* = 3). Groups (2.5 µM, 5 µM, 7.5 µM and 10 µM ) were compared with negative control (NC) control, *p* > 0.05, one-way ANOVA with Tukey’s multiple comparisons test. (**C**) LDH release quantity assay in HSC-T6 cells treated with Dot1l at different concentrations; 0.1% TritonX-100 was used as positive control. Data are presented as means ± SEM (*n* = 3). Groups (2.5 µM, 5 µM, 7.5 µM and 10 µM) were compared with NC control, *p* > 0.05, one-way ANOVA with Tukey’s multiple comparisons test.

To evaluate cell viability and growth after Dot1l peptide treatment, an MTT assay was conducted on HSC-T6 and MCF7 (Figure 5A) cells treated with an indicated range of Dot1l peptide concentrations. The assay was performed after 24 h and then 48 h later. As shown in Figure 5A, no significant inhibition of indicated HSC-T6 and MCF7 cell growth was observed, which suggested that Dot1l peptide treatment was safe and non-cytotoxic. Afterwards, classic hemolysis assay (Figure 5B) and LDH assays (Figure 5C and Supplementary Figure S4A) were conducted. Moreover, cell cycle (Supplementary Figure S4B) and cell apoptosis (Supplementary Figure S4C) examined by flow cytometry did not show significant differences between control and Dot1l peptide treatment. Thus, these data indicate no membrane disturbance in red blood cells or cultured HSC-T6 and MCF7 cells.

### 3.6. Dot1l-Mediated DNA Delivery

**Figure 6:**
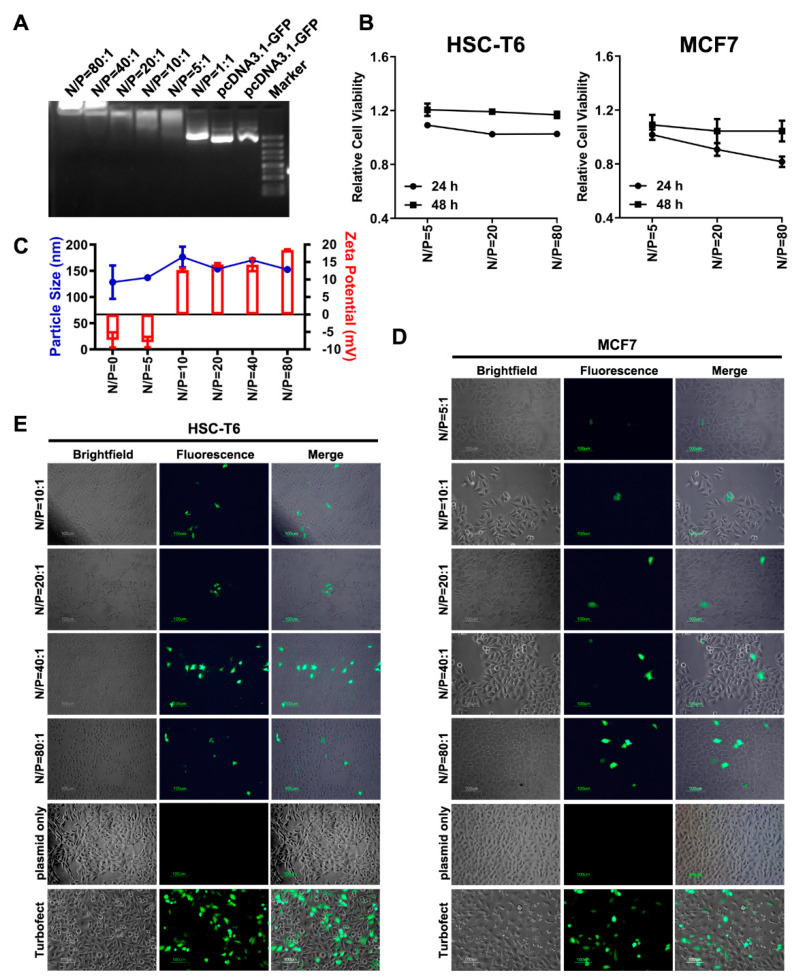
Dot1l-mediated plasmid delivery. (**A**) Gel retardation assay on different N/P ratios of Dot1l peptide and pcDNA3.1-GFP. (**B**) Cell viability of HSC-T6 and MCF7 cells treated with Dot1l peptide and pDNA examined by MTT assay. Data are presented as means ± SEM (*n* = 3), *p* > 0.05, two-way analysis of variance (ANOVA) with Bonferroni’s multiple comparison test. (**C**) Zeta-potentials and average particle size of Dot1l/pDNA complexes at various N/P ratios. Blue line indicates particle size; red bar indicates zeta potential. (**D**) Fluorescence microscopy of GFP expression in MCF7 cell transfected by Dot1l/pDNA complex at various N/P ratios after 24 h. (**E**) Fluorescence microscopy of GFP expression in HSC-T6 cell transfected by Dot1l/pDNA complex at various N/P ratios after 24 h.

The data shown above suggest that Dot1l can penetrate cells based on the positive charge-rich residue characteristics. However, Dot1l’s ability to mediate negatively charged materials, such as DNA, into cells is still unknown. Before evaluating Dot1l-mediated plasmid DNA (pDNA) delivery, agarose gel retardation analysis was conducted to assess their interactions. Results shown in Figure 6A suggests that Dot1l peptide-pDNA complex interactions occur at the N/P ratio of 5:1. In a ratio of 80:1, no migration of the peptide-pDNA complex occurred. We also examined the stability of peptide-pDNA complex in 50% serum (Supplementary Figure S5A). Our data suggest that the peptide-pDNA complex was stable in the serum solution even after 4 h of incubation. Then, the cytotoxicity evaluation of the Dot1l/pDNA complex was conducted using MTT assay. Over 80% of HSC-T6 cells and 90% of MCF7 cells were viable and grew well after peptide-pDNA complex treatment (Figure 6B). Electrostatic interactions examined by zeta-potential and particle size of Dot1l peptide/pDNA were conducted. Figure 6C shows that the charge of Dot1l peptide/pDNA was neutralized between N/P = 5 and N/P = 10. Zeta values of peptide/DNA complex increased as the N/P ratios elevated, while the particle size of Dot1l peptide/pDNA complex was between 150 nm and 100 nm in diameter. Furthermore, Dot1l peptide-based pDNA transfection efficiencies were evaluated against HSC-T6 and MCF7 cells after 24 h (Figure 6D,E, and Supplementary Figure S5B) and 48 h (Supplementary Figure S5C,D) of transfection. GFP expression was examined and images were captured by fluorescence microscopy. GFP expression in MCF7 (Figure 6D) and HSC-T6 (Figure 6E) could be detected from the N/P ratio of 5:1 to 80:1. GFP expression decreased in HSC-T6 and MCF7 cells after 48 h of transfection, resulting in the loss of plasmid content after cell division (Supplementary Figure S5C,D). Compared with common transfection reagent Turbofectin, the efficiency of peptide-based transfection was slightly weaker (Supplementary Figure S5B). Collectively, data reported here confirm that electrostatic interaction of Dot1l peptide/pDNA complex could be formed and did not induce unacceptable cytotoxicity to cells examined, while efficiently mediating pDNA transfection in vitro.

### 3.7. Dot1l Peptide Mediate Macromolecule GFP Protein Delivery In Vitro

**Figure 7:**
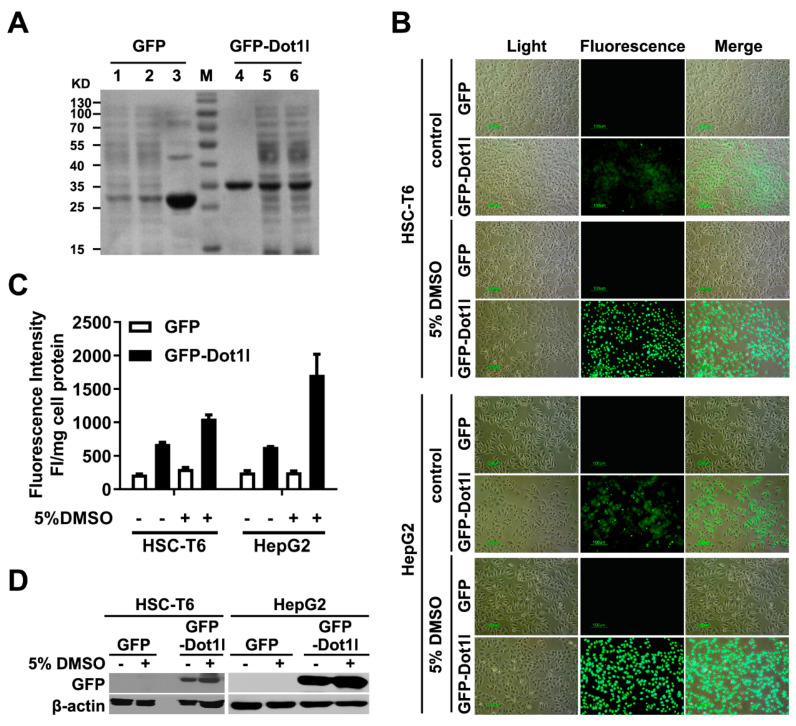
(**A**) Coomassie Brilliant Blue-stained SDS-PAGE of GFP-Dot1l fusion protein produced by recombinant prokaryotic expression system. Lanes 1 and 6 indicate no isopropyl-β-D-thiogalactopyranoside (IPTG) induction, lanes 2 and 5 indicate IPTG induction, lanes 3 and 4 indicate purified protein. (**B**) Fluorescence microscopy of purified GFP and GFP-Dot1l protein delivery in HSC-T6 and HepG2 cells with or without DMSO pretreatment. (**C**) Fluorescence intensity quantification of purified GFP and GFP-Dot1l protein in the cytosol. The statistical analysis is shown in Supplementary Figure S1F (top panel indicates HSC-T6, bottom panel indicates HepG2). (**D**) GFP and GFP-Dot1l protein in the cytosol of HSC-T6 and HepG2 cells examined by Western blotting; anti-GFP antibody was used to detect the protein. Cell lysate fluorescence intensity was adjusted by protein concentration examined by bicinchoninic acid (BCA) assays.

The aforementioned data suggest that Dot1l can enter into the cells and allow efficient transfection. After compaction with pDNA, we fused the Dot1l peptide coding sequence with the GFP sequence, and generated the pET15b-GFP-Dotl1 prokaryotic expression plasmid. Recombinant proteins produced and purified from *Escherichia coli* and the fusion protein before and after purification were examined by SDS-PAGE (Figure 7A). HSC-T6 and HepG2 cells were treated with GFP-Dot1l fusion protein with or without DMSO treatment. GFP-Dot1l fluorescence was distributed well in the cytoplasm of HSC-T6 and HepG2 cells (Figure 7B) and the uptake efficiency of GFP-Dot1l was quantified (Figure 7C, Supplementary Figure S1E). GFP-Dot1l fusion protein in the cytosol was also examined by Western blot (Figure 7D). These results suggest that Dot1l peptide can mediate macromolecule GFP protein with detectable delivery in vitro.

## 4. Discussion

Owing to the progress of molecular biology and gene engineering, numerous genes or proteins can be considered a therapeutic to treat otherwise incurable diseases. However, very poor cell membrane permeability limits the number of delivery methods, thus preventing their application due to low cellular uptake efficiency. Nonetheless, facilitated delivery involving peptide-mediated delivery holds great potential because peptides can be easily synthesized or recombinantly expressed in an *E. coli* expression system. From a functional and safety perspective, peptide sequences originate from native protein precursors, which are deliberately designed and engineered to be biocompatible peptides. These peptides can be specifically bound with high affinity to their cognate receptors, which are sometimes comparable to those of full-length antibodies. Therefore, numerous reports showing TAT and other CPPs have been widely used for therapeutic nucleic acid delivery. Still, challenges remain, and improvement exists on many fronts, such as the efficiency and targeting of CPP.

In this study, we found that a short peptide derived from Dot1l protein rich in basic residues has a higher penetration efficiency than classic CPP-TAT and shows good penetrating property in different cell lines. It can enter cells in a concentration-dependent manner; its penetration efficiency can be prompted by DMSO pretreatment. Penetration of Dot1l does not show cell specificity, and may be used as a common tool for the delivery. Although the exact mechanism of Dot1l penetration is unknown, our data suggest that it may directly translocate to the cytosol. Our data also show that endocytosis is not the major pathway of Dot1l penetration because endocytosis-related inhibitor treatment can not completely inhibit its penetration. Moreover, from the established web server prediction, the data suggests that Dot1l peptide can interact with the membrane, albeit the peptide-membrane interaction was weak. Lastly, not only can Dot1l enter into cells itself, but it can also efficiently mediate non-covalently conjugated plasmid and covalently conjugated GFP protein into cell specific compartments. Dot1l also maintains cargo function.

At present, the exact mechanism of CPP translocation is still not well understood. The 21-residue peptide TP10 (transportan 10), derived from mastoparan [33,34,35], has a high proportion of arginine and/or lysine. Dot1l is also lysine- and arginine-rich, as demonstrated by the structure of Dot1l peptide predicted by different web servers clearly showing an α-helical structure. This Dot1l structure is similar to TP10, forming α-helices. However, because of this, Dot1l cannot be inserted into the membrane to induce cell membrane permeability by forming pores, which was supported by our hemolysis and LDH releasing data that indicated no content was released intracellularly from red blood cells, as well as in vitro cultured cells. Thus, our results demonstrate that Dot1l has to directly cross the plasma membrane, similarly to other CPPs such as YopM [36,37].

## 5. Conclusions

In summary, the Dot1l peptide may represent a promising CPP. Dot1l can enter into a variety of cells with or without help of penetration enhancers. Its major penetration pathway involves direct translocation, and it does not induce cytotoxicity. Moreover, Dot1l can not only permeate into cells itself, but can also efficiently mediate non-covalently conjugated nucleic acid and covalently conjugated fusion protein.

Regenerative medicines can address the underlying cause of disease, and thus hold enormous potential for a variety of currently uncurable conditions with high unmet clinical need. However, the lack of adequate safety and efficacy of delivery tools to generate patient-specific induced pluripotent stem cells (iPSCs) is currently limiting regenerative medicine-based therapies. The new findings in our manuscript make Dot1l attractive as a potential novel delivering tool for drug development, innovative real time-FACS (flow cytometry cell sorting) [38], regenerative medicine [1], gene therapy, and gene editing-based therapy [4].

## Supplementary Materials

The following are available online at http://www.mdpi.com/2218-273X/10/2/217/s1, Figure S1: Statistical analysis. (A) Corresponds to Figure 2B. (B) Corresponds to Figure 2D w/o DMSO pretreatment. (C) Corresponds to Figure 2D of 5% DMSO pretreatment. (D) Corresponds to Figure 3B. (E) Corresponds to Figure 3D. (F) Corresponds to Figure 7C. Figure S2: Penetration efficiency of Dot1l in different conditions. (A) Quantification of Dot1l penetration with or without TB treatment. (B) Effects of different incubation times on the Dot1l penetration. (C) Penetration efficiency of Dot1l in different cell lines. (D) Comparison of penetration efficiency from different CPPs in MCF7 and HSC-T6 cells. Figure S3: Prediction of different CPP-membrane interactions in aqueous and lipid bilayer environments. Figure S4: Dot1l safety examined by LDH release, cell cycle, and cell apoptosis assay. (A) LDH release quantity assay in MCF7 cells treated with Dot1l at different concentrations. (B) Cell cycle examined by flow cytometry. (C) Cell apoptosis examined by flow cytometry with or without Dot1l incubation. Figure S5: Stability of Dot1l/pDNA complex and Dot1l peptide-mediated pDNA transfection. (A) Agarose gel examination of Dot1l peptide and pcDNA3.1-GFP complex at different N/P ratios in 50% serum solution. (B) Transfection efficiency quantification of Dot1l peptide/pDNA in different cell lines. (C) Dot1l peptide-mediated pDNA transfection in MCF7 cells (48 h) examined by fluorescence microscope. (D) Dot1l peptide-mediated pDNA transfection in HSC-T6 cells (48 h) examined by fluorescence microscope.
