# Correction: Geng et al. Intracellular Delivery of DNA and Protein by a Novel Cell-Permeable Peptide Derived from DOT1L. *Biomolecules* 2020, *10*, 217

**DOI:** 10.3390/biom14101199

**Published:** 2024-09-24

**Authors:** Jingping Geng, Xiangli Guo, Lidan Wang, Richard Q. Nguyen, Fengqin Wang, Changbai Liu, Hu Wang

**Affiliations:** 1 Department of Pathology and Immunology, Medical School, China Three Gorges University, Yichang 443002, China; gjp081188@outlook.com (J.G.); biomed-xiangli-guo@outlook.com (X.G.); wang473119037@outlook.com (L.W.); 2 Hubei Key Laboratory of Tumor Microenvironment and Immunotherapy, China Three Gorges University, Yichang 443002, China; wfq1248869512@outlook.com; 3 Institute for Cell Engineering, Johns Hopkins University School of Medicine, Baltimore, MD 21205, USA; nguyen.richard15@gmail.com

In the original article [1], there were errors in Figure 2A,C, Figure 3A,C and Figure S2C. We noticed that the 37 °C group without DMSO in Figure 3A was duplicated from Figure 2A (the 5 µM group), and Figure 3C (the control group) was duplicated from Figure 2C (the TB (-) group, without DMSO). We found that the light and fluorescence images from Figure 2A (the 7.5 µM group, 20×), Figure 2A (the 10 µM group, 20×), and Figure S2C (the DMSO-treated HSC-T6 group, 20×) were mismatched separately. These were unconscious mistakes during figures processing.

Corrections have been made to the images in Figure 3A (the 37 °C group, without DMSO) and Figure 3C (the control group). The mismatched light and fluorescence images of Figure 2A (the 7.5 µM group, 20×, light panel), Figure 2A (the 10 µM group, 20×, fluorescent panel), and Figure S2C (the DMSO-treated HSC-T6 group, 20×, light panel) have been corrected. The corrected figures are presented below. 

**Figure 2:**
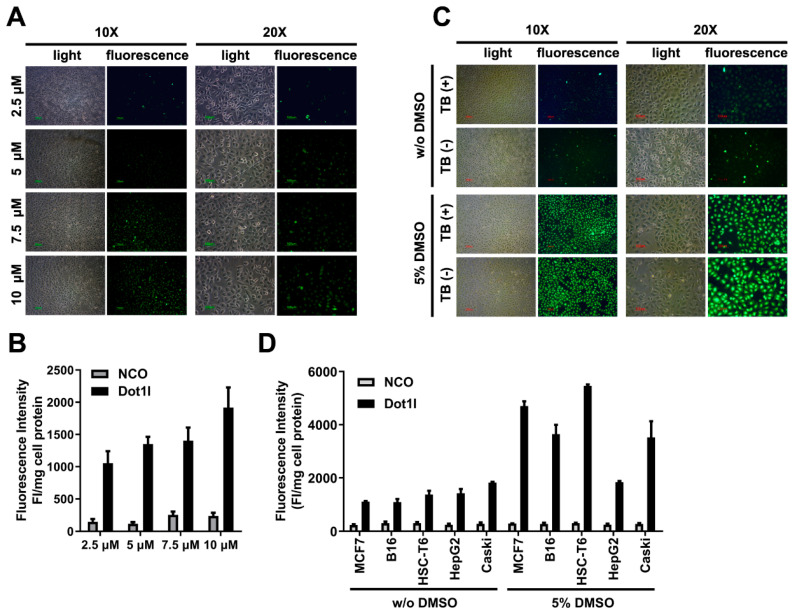
Fluorescence labelled Dot1l peptide penetration in cultured cells. (**A**) Fluorescence microscopy of fluorescein isothiocyanate (FITC)-labeled Dot1l peptide with different concentrations. (**B**) Quantization analysis of FITC-labeled Dot1l peptide corresponding to fluorescence microscopy with different concentration measurements. The statistical analysis is shown in Supplementary Figure S1A. (**C**) Fluorescence microscopy of FITC-labeled Dot1l peptide with or without trypan blue incubation in the DMSO-pretreated or control group. (**D**) FITC-labeled Dot1l peptide penetration in different cell lines (MCF7, B16, HSC-T6, Caski, and HepG2) with or without DMSO pretreatment. The statistical analysis is shown in Supplementary Figure S1B,C. Cell lysate fluorescence intensity was adjusted by protein concentration examined by Bradford assay.

**Figure 3:**
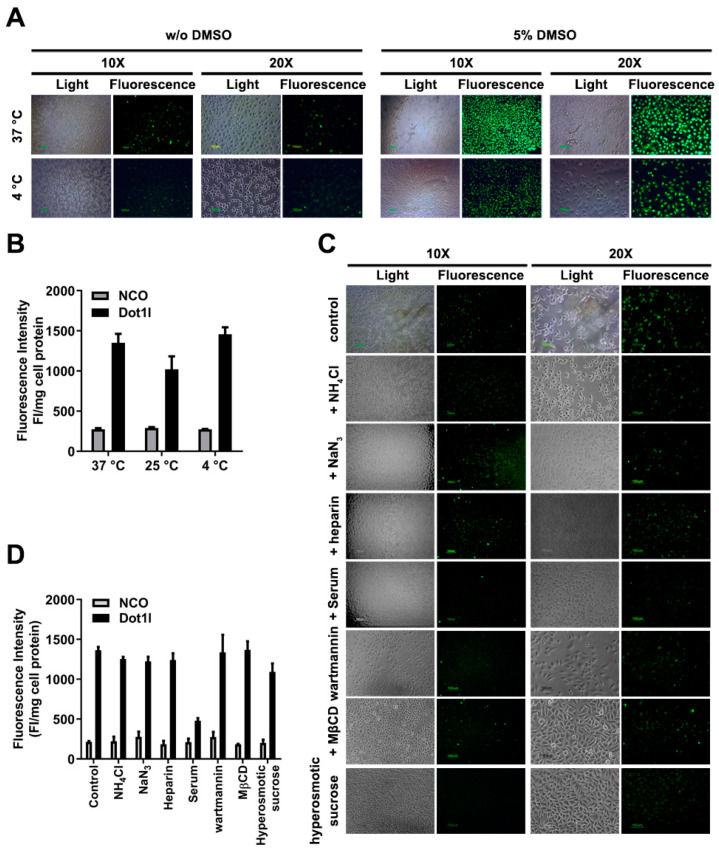
Effects of Dot1l peptide penetration under different conditions. (**A**) Fluorescence microscopy of FITC-labeled Dot1l peptide at 37 °C and 4 °C. (**B**) Quantization of Dot1l peptide penetration in MCF7 cells at different temperatures. Data are presented as means ± SEM (*n* = 3). The statistical analysis is shown in Supplementary Figure S1D. (**C**) Fluorescence microscopy of different inhibitors’ exposure on Dot1l peptide penetration. (**D**) Suppression of different inhibitors’ exposure on Dot1l peptide penetration. Data are presented as means ± SEM (*n* = 3). The statistical analysis is shown in Supplementary Figure S1E. Cell lysate fluorescence intensity was adjusted by protein concentration examined by Bradford assay.

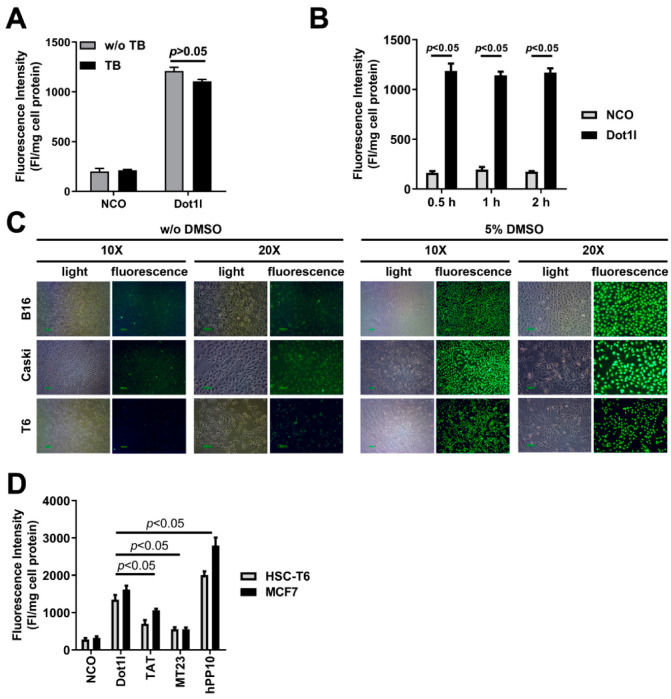

Figure S2 Penetration efficiency of Dot1l in different conditions.
A. Quantification of Dot1l penetration with or without TB treatment.
B. Effects of different incubation time to the Dot1l penetration.
C. Penetration efficiency of Dot1l in different cell lines.
D. Comparison of penetration efficiency from different CPPs in MCF7 and HSC-T6 cells.

The authors apologize for any inconveniences caused and state that the scientific conclusions of the paper are unaffected. This correction was approved by the Academic Editor. The original publication has also been updated.
